# Systematized water content calculation in cartilage using T1-mapping MR estimations: design and validation of a mathematical model

**DOI:** 10.1007/s10195-016-0433-8

**Published:** 2016-10-22

**Authors:** J. M. Shiguetomi-Medina, J. L. Ramirez-GL, H. Stødkilde-Jørgensen, B. Møller-Madsen

**Affiliations:** 1 Orthopaedic Research Laboratory, Aarhus University Hospital NBG, Aarhus University, Noerrebrogade 44 Building 1A, 8000 Aarhus C, Denmark; 2 Department of Clinical Epidemiology and Public Health, Facultad de Medicina, Universidad Autónoma de San Luis Potosi, Venustiano Carranza 2045, 78210 San Luis Potosi, SLP Mexico; 3 The MR Research Center, Aarhus University Hospital, Skejby, Brendstrupgaardsvej 100, 8200 Aarhus N, Denmark; 4 Department of Children’s Orthopaedics, Aarhus University Hospital NBG, Noerrebrogade 44, 8000 Aarhus C, Denmark

**Keywords:** Water content, MR scanning, Mathematical model

## Abstract

**Background:**

Up to 80 % of cartilage is water; the rest is collagen fibers and proteoglycans. Magnetic resonance (MR) T1-weighted measurements can be employed to calculate the water content of a tissue using T1 mapping. In this study, a method that translates T1 values into water content data was tested statistically.

**Materials and methods:**

To develop a predictive equation, T1 values were obtained for tissue-mimicking gelatin samples. 1.5 T MRI was performed using inverse angle phase and an inverse sequence at 37 (±0.5) °C. Regions of interest were manually delineated and the mean T1 value was estimated in arbitrary units. Data were collected and modeled using linear regression. To validate the method, articular cartilage from six healthy pigs was used. The experiment was conducted in accordance with the Danish Animal Experiment Committee. Double measurements were performed for each animal. Ex vivo, all water in the tissue was extracted by lyophilization, thus allowing the volume of water to be measured. This was then compared with the predicted water content via Lin’s concordance correlation coefficient at the 95 % confidence level.

**Results:**

The mathematical model was highly significant when compared to a null model (*p* < 0.0001). 97.3 % of the variation in water content can be explained by absolute T1 values. Percentage water content could be predicted as 0.476 + (T1 value) × 0.000193 × 100 %. We found that there was 98 % concordance between the actual and predicted water contents.

**Conclusion:**

The results of this study demonstrate that MR data can be used to predict percentage water contents of cartilage samples.

**Level of evidence:**

3 (case-control study).

## Introduction

A mathematical model can be defined as a mathematical representation of the behavior of a real device or object [1]. In science, it is essential to model phenomena because these models can be used to explain behavior, thus allowing predictions to be made [2]. Cartilage is an intricate balance of water, chondrocytes, and a rich extracellular matrix of collagen fibers and proteoglycan molecules. Up to 80 % (by weight) of this tissue is water [3]. As cartilage is mainly composed of water, the water content of the cartilage is an indicator of cartilage health. Using magnetic resonance (MR), it is possible to accurately describe morphology and tissue integrity. Some authors consider this technique the new gold standard for cartilage image analysis [3, 4]. It has been reported that disease affects the water content of cartilage even before clinical or radiological signs appear [5]. Therefore, it is important to quantify the water content in this tissue as the water content can then be used to diagnose disease and estimate its prognosis [6]. T1-weighted magnetic resonance measurements provide enough data to allow the water content to be calculated. This method has been used in several scientific fields; see for instance [7–10], although these reports only provide information on the intensity of the T1 signal. In the present study, a similar method was developed to quantify the T1 signal in cartilage [11]. To our knowledge, this is the first statistically proven method of translating T1-mapping values into clinically useful data (water content as a percentage or in milliliters) to be published. The aims of this study were therefore to develop an equation that could be used to estimate the percentage water content of cartilage tissue based on T1-weighted MR images, and to assess the performance of this equation in vivo.

## Materials and methods

### Development of the water content equation

**Fig. 1:**
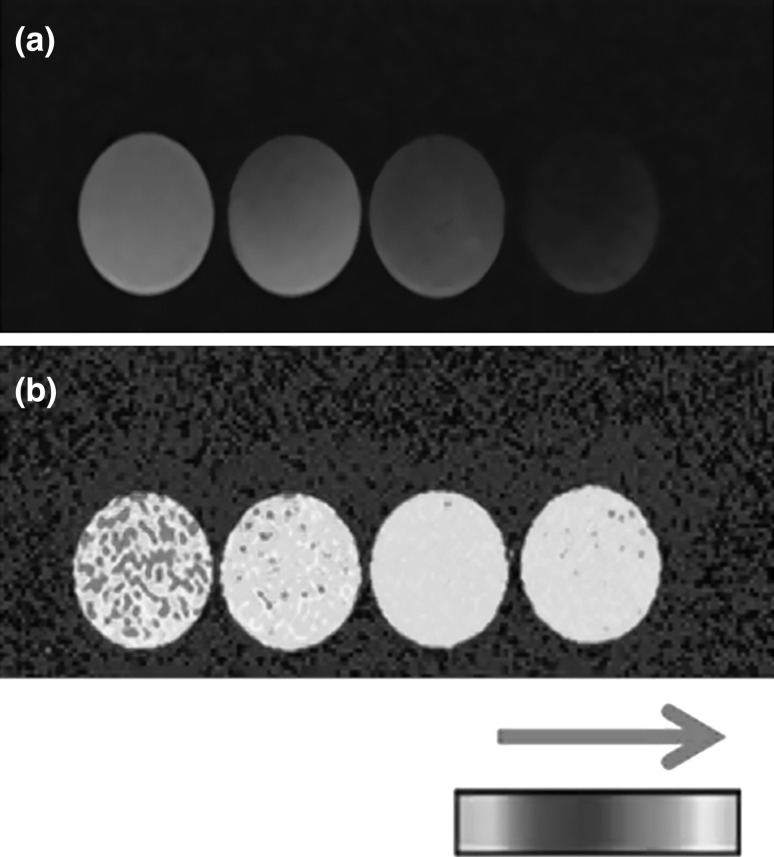
T1 signal of the water content in gelatine samples. Using T1-MR sequences, color heat maps of water content were obtained. **a** Absolute T1-value maps in gelatine samples with different water concentrations (*from* *left* *to* *right*: 100, 91, 80, 70 %). **b** The same samples in the inverse recuperation sequence are shown. The* arrow above the colorimetric scale* indicates increasing water content along the scale

T1 values were obtained from 45 tissue-mimicking gelatine samples (gelatine for microbiology, Merck, Darmstadt, Germany) with predetermined water concentrations (70, 75, 80, 85, 88, 91, 95, and 100 %). We analyzed the samples with a whole-body 1.5 T MRI scanner (Magnetom Avanto, Siemens, Erlangen, Germany). Absolute T1 values were calculated in real maps using inverse angle phase (inverse sequence echo gradient of 1–5°, 2–30°, ET 1.61 ms, RT 15 ms, image thickness 4 mm, NEX 3, FOV 180 × 101 mm in a 256 × 256 pixel matrix), and with inverse sequence recuperation (11 inversion times from 200 to 2200 ms, image thickness 4 mm, FOV 200 × 200 mm in a 256 × 256 pixel matrix) at a constant 37 (±0.5) °C. Images acquired this way were heat maps with color values representing different water concentrations (Fig. 1). The region of interest in the field of view was manually delineated and the mean T1 value of each individual voxel was estimated using the software Siswin v.0.9 (Ringaard S, 2008). Data were collected and then modeled via linear regression by fitting the water content values to the T1 values.

### Concordance between the predicted and real water contents

To assess the performance of the predictive equation for a sample of healthy cartilage, the tibial proximal growth plates of six healthy, skeletally immature, 30-kg Danish Landrace pigs were used. The experiment was conducted in accordance with the Danish Animal Research Guidelines, and was accepted and authorized by the Danish Animal Experiment Committee (study J. No. 2008/561-329). The animals received an intramuscular injection of ketaminol (5 mg/kg* S*-ketamine, Pfizer, Berlin, Germany) and midazolam (0.5 mg/kg, Hypnomidate, Janssen–Cilag, Beerse, Belgium). The animals were intubated and connected to a ventilator (frequency rate 16, volume 400 ml, 30 % oxygen). Anesthesia was maintained with intravenous infusion of propofol (5 mg/kg/h, Fresenius Kabi AB, Uppsala, Sweden) and fentanyl (0.025 mg/kg/h, Haldid, Janssen–Cilag). Measurements were performed in both hind legs of each animal on the same scan using the MR protocols discussed above. The area of interest was manually delineated on the growth plate and the mean T1 values were obtained. The T1 values were inputted into the water content equation, and the estimated percentage water content was calculated. Right after euthanasia, the tibiae were harvested and the proximal growth plates were isolated. All water in the tissue was extracted from each growth plate by lyophilization (freeze-drying), allowing us to measure the actual volume of water in the tissue. The difference between the wet and dry weights was used to estimate the percentage water content. The actual water content was compared to the predicted water content using Lin’s concordance correlation coefficient, which has been shown to be superior to other correlation coefficients in paired data analysis [12, 13]. The statistical package R v.3.0.2 was used [14] and the 95 % confidence level was derived.

## Results

### Water content equation

**Fig. 2:**
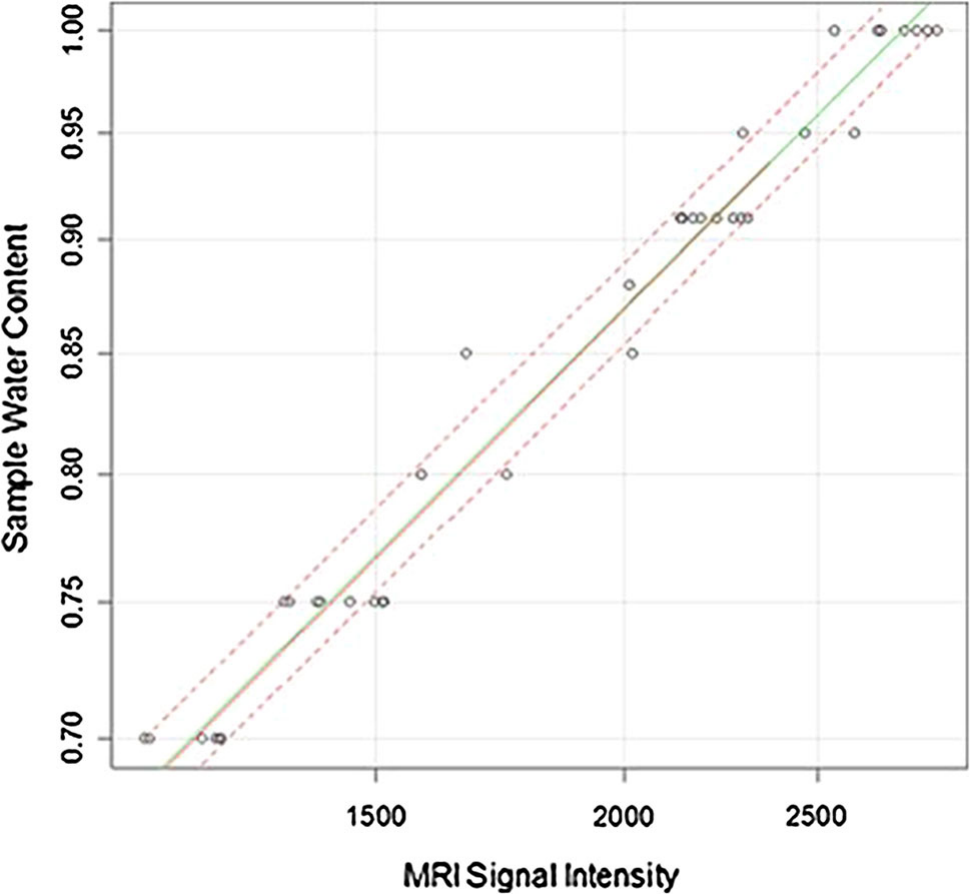
Statistical model for the development of the water content prediction equation. T1 values were plotted against the corresponding known water content values. The *continuous red line* represents the line of best fit, the *continuous green line* shows the bootstrap validation. *Dotted lines* represent 95 % confidence intervals (color figure online)

The developed mathematical model gave a better fit (with high statistically significance) to the known water content values than a null model did (*p* < 0.0001, *r* ^2^ = 0.973). It was adjusted by 150 bootstrap repetitions to an *r* ^2^-corrected index of 0.9715 (Fig. 2). In the equation, when the mean absolute T1 value increases by one unit, the water content increases by 0.019 % (95 % CI 0.018–0.020, *p* < 0.0001). The final equation for the predicted percentage water content was: % water content = (0.476 + T1 signal intensity × 0.000193) × 100 %.

### Water content concordance

**Fig. 3:**
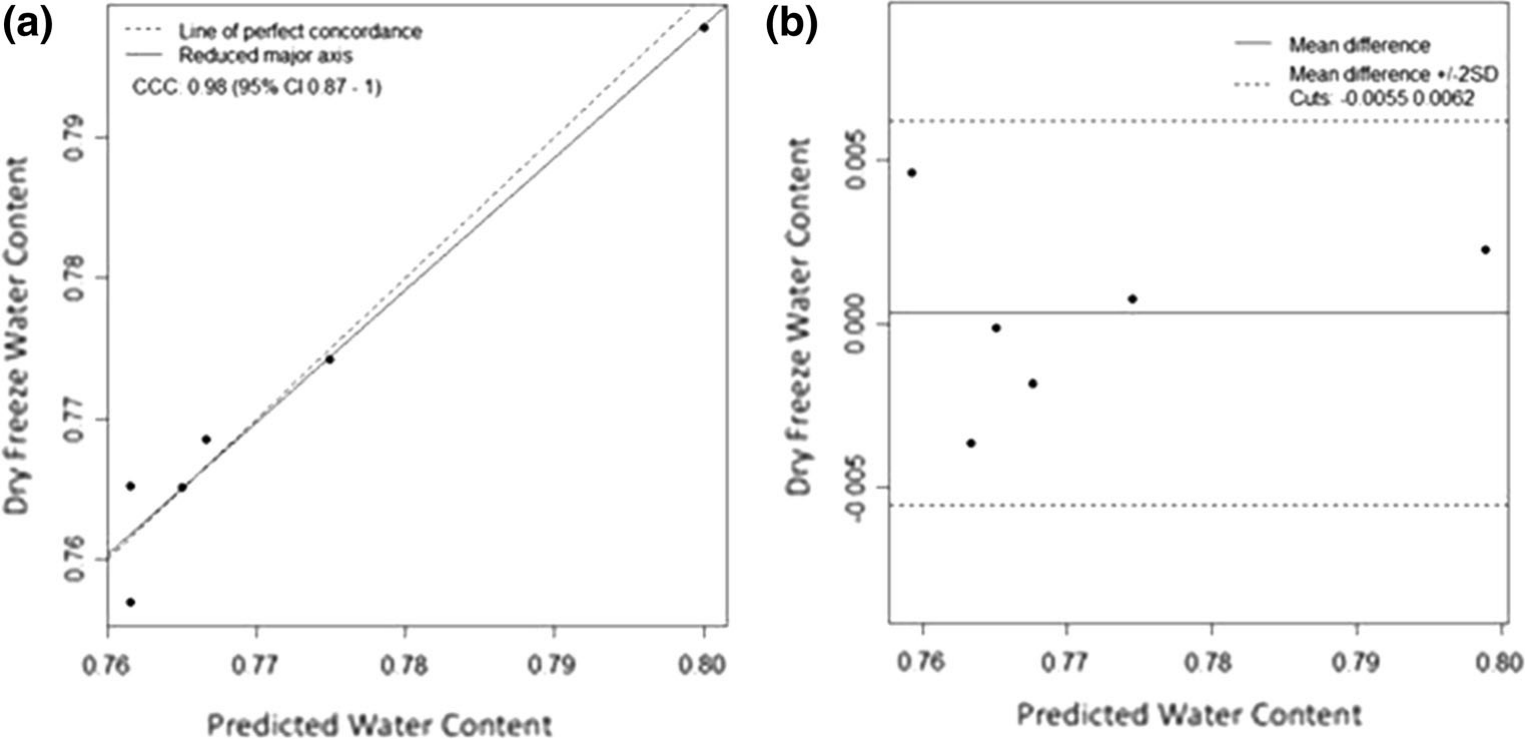
Validation of the predictive equation using actual cartilage water contents. **a** Lin’s concordance correlation coefficient for a comparison of the predicted water content to the freeze-dried water content was obtained based on six samples. **b** Bland–Altman plot for the same samples

Upon comparing the predicted pig physis water contents with the corresponding actual water contents, a Lin’s concordance correlation coefficient of 0.98 (95 % CI 0.87–1.0) was obtained (Fig. 3).

## Discussion

The evaluation of cartilage using magnetic resonance data provides information on the integrity and physiological condition of tissue; specifically, its water content [11]. Our results allow the T1 value of any cartilage sample to be converted into a percentage water content through the use of a predictive equation. We believe that such an approach is useful in the clinical arena since it provides data that can be easily handled and translated into clinically relevant information. T1 values provide information on the water content because the protons in the cartilage interact with the water molecules that surround them [15]. Since this lattice consists mainly of water [3], using inverse sequence echo gradients allows the free water component to be differentiated from the protein-bound water component [15]. The content of free water is an indicator of the health of the tissue [5]. Because absolute T1 values are considered in this approach, the predictive equation could be used for values obtained under other magnetic fields (we performed our experiments under a 1.5 T magnetic field). However, a specific experiment to prove this should be conducted. The predictive equation developed in this study was shown in the in vivo assays to predict the true water content with 98 % accuracy. The clinical significance of the quantification of cartilage water content is the early identification of patients who present degeneration of the proteoglycan matrix as well as tissue hydration even before clinical symptoms occur. This could allow the development of predictive parameters for cartilage health and the establishment of early therapy in the future. The main limitation of this study is that a small sample of real tissue was used for the correlation assay. Although our results appear to be robust, they cannot be used to make clinical predictions. Clinical implications of this technique may be the assessment of subclinical damage in joint cartilage, the assessment of water content in intervertebral discs, and the development of prognostic indicators for osteoarthritis and cartilage injuries. Gelatine samples resemble not only the cartilage lattice but also other tissues with high water concentrations, meaning that this equation could be applied to those tissues too, thus allowing the identification of other diseases, although further studies must be carried out to verify this. They may be multiple applications of this approach beyond the field of musculoskeletal diseases, and tests assessing whether the predictive equation is useful in tissues with different lattices should be performed. Potential applications of this technique could be the quantification of cerebral edema in patients with traumatic brain injury, the quantification of water volume in patients with edematous states, and the estimation of blood volume in a tumor. Also, most newly diagnosed patients are not in the early stages of disease. This equation could be used in an objective follow-up to treatment or disease progression. Many orthopedic diseases involve cartilage; this equation could help with diagnosis and treatment. The utilization of the equation must be approved by the treating orthopedic surgeon. We are proposing a new tool.

In conclusion, T1 values of cartilage obtained through inverse angle phase and reverse sequence retrieval methods provide information on the water content of tissue. These values can be translated into percentage water contents using a predictive formula with an in vivo accuracy of 98 %.
